# Demyelinating Syndromes in Systemic Lupus Erythematosus: Data From the “Attikon” Lupus Cohort

**DOI:** 10.3389/fneur.2022.889613

**Published:** 2022-05-11

**Authors:** Dionysis Nikolopoulos, Dimitrios Kitsos, Matilda Papathanasiou, Noemin Kapsala, Panagiotis Garantziotis, Antigone Pieta, Ourania Gioti, Alexandros Grivas, Konstantinos Voumvourakis, Dimitrios Boumpas, Antonis Fanouriakis

**Affiliations:** ^1^Rheumatology and Clinical Immunology Unit, 4th Department of Internal Medicine, Attikon University Hospital, Joint Rheumatology Program, National and Kapodistrian University of Athens Medical School, Athens, Greece; ^2^Department of Neurology, Attikon University Hospital, School of Medicine, National and Kapodistrian University of Athens, Athens, Greece; ^3^Department of Radiology, Attikon University Hospital, National and Kapodistrian University of Athens, School of Medicine, Athens, Greece; ^4^Department of Rheumatology, “Asklepieion” General Hospital, Athens, Greece; ^5^Department of Propaedeutic Internal Medicine, Medical School National Kapodistrean University of Athens Medical School, Athens, Greece

**Keywords:** systemic lupus erythematosus, multiple sclerosis, demyelination, central nervous system, outcome demyelination in systemic lupus erythematosus

## Abstract

**Background:**

The demyelinating syndromes of the central nervous system (CNS) that occur in the context of systemic lupus erythematosus (SLE) may represent a manifestation of neuropsychiatric lupus (NPSLE) or an overlap of SLE and multiple sclerosis (MS). The differential diagnosis between the two entities has important clinical implications because the therapeutic management differs.

**Objectives:**

To characterize CNS demyelinating syndromes in a large SLE cohort as neuropsychiatric SLE (NPSLE) or SLE-MS overlap using a multidisciplinary approach and existing diagnostic (for MS) and classification criteria (for SLE).

**Methods:**

Patients from the “Attikon” lupus cohort (*n* = 707) were evaluated for demyelinating syndromes. Clinical, laboratory, and neuroimaging data were recorded for each patient. Following multidisciplinary evaluation and application of criteria, the demyelinating syndrome was attributed to either SLE or MS. Patients with transverse myelitis were not included in this study.

**Results:**

We identified 26 patients with demyelinating syndromes (3.7%). Of them, 12 were diagnosed as primary SLE-demyelination (46.2%) and 14 as overlap SLE-MS (53.8%). The two groups did not differ with respect to rheumatologic and neurologic manifestations or autoantibodies. SLE patients with demyelination manifested mild extra-CNS disease mainly involving joints and skin, while severe non-CNS manifestations were rare. However, these patients were less likely to have elevated IgG index (OR 0.055 95% CI: 0.008–0.40) and positive oligoclonal bands (OR 0.09 95% CI: 0.014–0.56), as well as brain lesions in the spinal cord, infratentorial, periventricular, and juxtacortical regions. A single brain region was affected in 9 patients with SLE-demyelination (75%), while all patients with MS-SLE had multiple affected brain regions. MS-SLE overlap was associated with an increased likelihood of neurologic relapses (OR 18.2, 95% CI: 1.76–188), while SLE-demyelination patients were less likely to exhibit neurological deficits (EDSS >0) at the last follow-up visit (50 vs. 78.6% in SLE-MS, respectively).

**Conclusions:**

Demyelination in the context of SLE follows a more benign course compared to a frank SLE-MS overlap. Extension of follow-up will ascertain whether patients with SLE-demyelination evolve to MS, or this is a *bona fide* NPSLE syndrome.

## Introduction

Among the many neuropsychiatric syndromes that constitute neuropsychiatric systemic lupus erythematosus (NPSLE), demyelinating syndrome (SLE-DS), termed lupoid sclerosis in the past (1), is one of the most challenging and less well-studied. Indeed, the definition of SLE-DS, according to the 1999 American College of Rheumatology (ACR) nomenclature, (2) is almost indistinguishable from multiple sclerosis (MS), a prototype organ-specific autoimmune demyelinating disease. Both MS and SLE-DS require objective evidence of central nervous system (CNS) neurological dysfunction, with documentation of dissemination in space and time (multiple episodes and affected areas within the CNS). Moreover, for a diagnosis of MS to be established, old and revised diagnostic criteria mandate prior exclusion of other conditions that can better explain the clinical and paraclinical findings of an individual patient, with SLE being a fundamental alternative diagnosis. This complex reality often creates confusion in physicians who encounter lupus patients with a DS, regarding whether this represents a CNS manifestation of the disease or a mere segregation of two autoimmune diseases (which is far from uncommon in clinical practice) (3). This differential diagnosis affects also the therapeutic management because drugs that used to treat NPSLE and MS, excluding glucocorticoids, are largely different (4).

In a previous work, following a combined rheumatology–neurology longitudinal assessment, we characterized a cohort of patients who presented with DS with atypical features for MS, and who had clinical and/or serological evidence of a systemic autoimmune disease (5). We found that a significant proportion of patients presenting with demyelinating syndrome do not fulfill the criteria for MS after more than 3 years of follow-up, and frequently manifest features of a systemic autoimmune disease (like arthritis or inflammatory rashes), although not formally diagnosed with SLE. We coined the term “demyelination with autoimmune features (DAF)” to describe patients in this “gray area”.

As a follow-up to this work, and using the same multidisciplinary approach, we herein attempt to expand on these findings by providing a description of patients with SLE from the established “Attikon” lupus cohort who have experienced a DS without fulfilling the criteria for MS, and comparing them with patients from the same cohort who have been characterized as an overlap of SLE with MS. We undertook this study in an effort to identify similarities and differences, if any, between the two patient subgroups, and potentially identify parameters that may predict which lupus patients with DS will fulfill diagnostic criteria for MS during the course of follow-up.

## Patients and Methods

### Patients and Clinical Assessment

This study is part of a collaborative project initiated in 2016 between the Rheumatology and Clinical Immunology Unit and the Department of Neurology of “Attikon” University Hospital, Athens, aiming to evaluate patients presenting with a DS of immune origin (ie. excluding trauma/compression, ischemia, or demyelination due to metabolic derangements) for the presence of features of an underlying systemic autoimmune disease, mainly SLE. The methodology has been previously described (5); briefly, the two units established a mutual referral algorithm, including i) patients examined in the Department of Neurology with a DS not fulfilling criteria for MS who had features suggestive of a systemic autoimmune disease, and ii) vice versa, patients followed in the Rheumatology and Clinical Immunology Unit for a systemic autoimmune disease who later developed a DS.

The “Attikon” lupus cohort was established in 2015 in the Rheumatology Unit of the “Attikon” University Hospital, Athens, serving as a referral center for patients with lupus, as previously described (6, 7). As of December 2021, it includes 708 Caucasian SLE patients. The present study aimed to characterize CNS demyelinating syndromes within the “Attikon” lupus cohort as neuropsychiatric SLE or SLE-MS overlap, using the same multidisciplinary approach as above, including rheumatologic–neurologic and neuroradiologic evaluation. To this end, we reviewed all patients with SLE for underlying CNS demyelinating disease with respect to clinical and neuroimaging evidence. All patients with possible DS were referred for comprehensive neurological evaluation, including thorough clinical examination and laboratory tests, MRI of the CNS, and cerebrospinal fluid (CSF) analysis, including IgG index and screening for oligoclonal band. Exclusion criteria were a) patients with neuromyelitis optica spectrum disorders (NMOSD) or other primary CNS diseases, b) patients with longitudinal myelopathy spanning three or more vertebral bodies, and c) patients with CNS imaging findings more consistent with microischemic, rather than demyelinating lesions, as judged by an experienced neuroradiologist (MP).

Following inclusion in the study, patients with CNS demyelination were followed at regular visits in both rheumatology and neurology units, with documentation of new clinical, laboratory, and imaging data. At the last follow-up, patients fulfilling the criteria for MS were labeled as “overlap SLE/MS”, while DS not fulfilling the criteria for MS were diagnosed as “SLE-DS”.

### Definitions

Diagnosis of SLE was established by the American College of Rheumatology (ACR) 1997 and/or the Systemic Lupus Erythematosus International Collaborating Clinics (SLICC) 2012 criteria, combined with expert physician judgment (AF, DB). Similarly, the diagnosis of MS was established by 2010 McDonald criteria combined with expert physician judgment (DK, KV) (8). A clinically isolated syndrome (CIS) was defined as a single demyelinating attack without dissemination in time.

Neurological disability and severity at the last follow-up were assessed by the Expanded Disability Status Scale (EDSS) (9). Patients were categorized as having “mild,” “moderate,” or “severe” neurological disability at the previous visit based on EDSS score. Specifically, mild disability was defined as EDSS ≤ 2, while severe disability was defined as EDSS >4. Patients falling between these two definitions were classified as having moderate disability.

We also used the following definitions regarding response to treatment: (i) no response; neurological symptoms and disability remained stable or worsened during follow-up, (ii) partial response; neurological symptoms and disability improved but did not completely resolve, and (iii) complete response; no neurological symptoms and disability at last visit.

### Assessment of MRI

All MRIs were performed on 1.5 or 3 Tesla MR scanners and reviewed by an expert neuroradiologist (MP). Images included a standard clinical protocol for brain imaging with T1 pre- and post-contrast injection, T2 and fluid attenuated inversion recovery (FLAIR) sequences with gadolinium administration. For the most recent MRI of each patient, distribution of demyelinating lesions was divided into 5 regions: a) cortex, b) juxtacortical, c) periventricular, d) infratentorial, and e) spinal cord.

### Statistical Analysis

All captured data are stored electronically at “Attikon” Hospital. Descriptive statistics were undertaken for continuous variables, and mean (SD) or median (IQR) values were calculated for normally and non-normally distributed variables, respectively. Chi-square test and Student's *t*-test were used to compare categorical and continuous variables, respectively. Logistic regression was applied to calculate the odds ratio for categorical variables. For all comparisons, a *p*-value < 0.05 was considered statistically significant. All statistical analyses were performed using the Statistical Package for the Social Science (SPSS; SPSS Inc., Version 25.0, IBM Corp., Armonk, NY, USA). The study was approved by the Ethics Committee of the “Attikon” University Hospital of Athens, and patients provided informed consent for their participation (protocol number 103/06-03-2014).

## Results

### Demyelinating Syndromes in “Attikon” Lupus Cohort

From a total of 708 SLE patients in the “Attikon” cohort, we identified 26 patients with DS [3.7%, mean age at lupus diagnosis 46.9 (SD 12.3) years]. Median SLE disease duration at last visit was 60 months (IQR 52 months) and median follow-up since the onset of demyelination was 79 months (IQR 118 months). With all data available at the end of follow-up, of 26 patients, 12 were diagnosed as primary SLE-DS (final prevalence 1.7% of the SLE cohort) and 14 as overlap SLE-MS. At the end of follow-up, 5 of the 12 SLE-DS patients were diagnosed as CIS. In the majority of SLE-DS patients, first occurrence of a demyelinating event occurred following the diagnosis of lupus, while the majority of neurologic manifestations in SLE-MS overlap patients preceded SLE diagnosis (Supplementary Table 1).

### Demyelinating Syndrome in Lupus Is Associated Mild Disease Outside the CNS

Rheumatic clinical features and autoantibodies of the 26 SLE patients with DS are summarized in Table 1, in comparison to the remaining SLE cohort. Notably, SLE patients with demyelination tend to exhibit mainly musculoskeletal and mucocutaneous disease features; severe non-CNS manifestations were rarely observed in this patient subgroup. In addition, SLE-DS patients were less likely to be positive for specific lupus autoantibodies, although differences did not reach statistical significance. Additionally, rheumatic clinical manifestations and autoantibodies did not differ between SLE-MS and SLE-DS patients (Supplementary Table 2).

**Table 1:** Clinical features and autoantibodies in SLE patients with and without demyelinating syndromes.

**Clinical manifestations**	**SLE with demyelination (*n* = 26)**	**SLE (*n* = 681)**	***P*-value**
Acute cutaneous lupus, *n* (%)	24(92.3)	487(71.5)	**0.02**
Malar rash, *n* (%)	17(65.4)	325(47.7)	0.08
Photosensitivity, *n* (%)	8(30.8)	381(55.9)	**0.01**
Chronic cutaneous lupus *n* (%)	2(7.7)	76(11.2)	ns
Oral ulcers, *n* (%)	6(23)	189(27.8)	ns
Non-scarring alopecia, *n* (%)	6(23)	236(34.7)	ns
Inflammatory arthritis, *n* (%)	24(92.3)	581(85.3)	ns
Serositis, *n* (%)	2(7.7)	128(18.8)	0.15
Lupus nephritis, *n* (%)	1(3.8)	149(21.9)	**0.03**
Neuropsychiatric events*, *n* (%)	6(23)	109(16)	ns
Leukopenia, *n* (%)	4(15.4)	240(35.2)	**0.04**
Thrombocytopenia, *n* (%)	0(0)	116(17)	*ns*
Hemolytic anemia, *n* (%)	0(0)	24(3.5)	*ns*
Fever, *n* (%)	2(7.7)	223(33.6)	**0.007**
Raynaud's, *n* (%)	8(30.8)	260(38.2)	ns
**Autoantibodies**			
ANA, *n* (%)	24(92.3)	658(96.6)	ns
Anti-dsDNA, *n* (%)	7(26.9)	285(41.6)	ns
Anti-Smith, *n* (%)	2(7.7)	52(7.6)	ns
Low C3 and/or C4, *n* (%)	12(46.2)	329(48.3)	ns
Anti-SSA, *n* (%)	7(26.9)	180(26.4)	ns
Anti-SSB, *n* (%)	3(11.5)	72(10.6)	ns
Anti-phospholipids, *n* (%)	3(11.5)	181(26.6)	0.08
Anti-RNP, *n* (%)	3(11.5)	60(8.8)	ns

* *Excluding demyelinating events. Values in bold represent comparisons that reached statistical significance (P < 0.05)*.

### Patients With SLE-MS Overlap Display Intrathecal Immunoglobulin Production and a Higher Burden of MRI Lesions in the CNS

Similar to rheumatic clinical manifestations, no significant differences were observed between patients with SLE-DS and SLE-MS in terms of neurologic manifestations (Supplementary Table 3). However, CSF and imaging findings differed between the two groups. Notably, patients with SLE-DS were significantly less likely to have an elevated IgG index (OR 0.05 95% CI: 0.008–0.40) and positive oligoclonal bands in the CSF (OR 0.09 95% CI: 0.014–0.56). More specifically, no patient with SLE-DS tested positive for type II oligoclonal bands, indicative of purely intrathecal immunoglobulin production, contrary to SLE-MS overlap patients who were predominantly positive for type II oligoclonal bands (Table 2).

**Table 2:** Cerebrospinal fluid findings of patients with SLE-demyelinating syndromes compared to SLE-MS.

	**Total (*n* = 26)**	**SLE-DS (*n* = 12)**	**SLE-MS (*n* = 14)**	***p*-value**
IgG index >0.65, *n* (%)	15 (57.7)	3 (25.0)	12 (85.7)	0.002
Positive oligoclonal bands, *n* (%)	24 (53.8)	3 (25.0)	11 (78.6)	0.006
Type II	8	0	8	NA
Type III	4	1	3	0.35
Type IV	2	2	0	NA

*NA, Not applicable due to zero values in one of comparators*.

**Figure 1:**
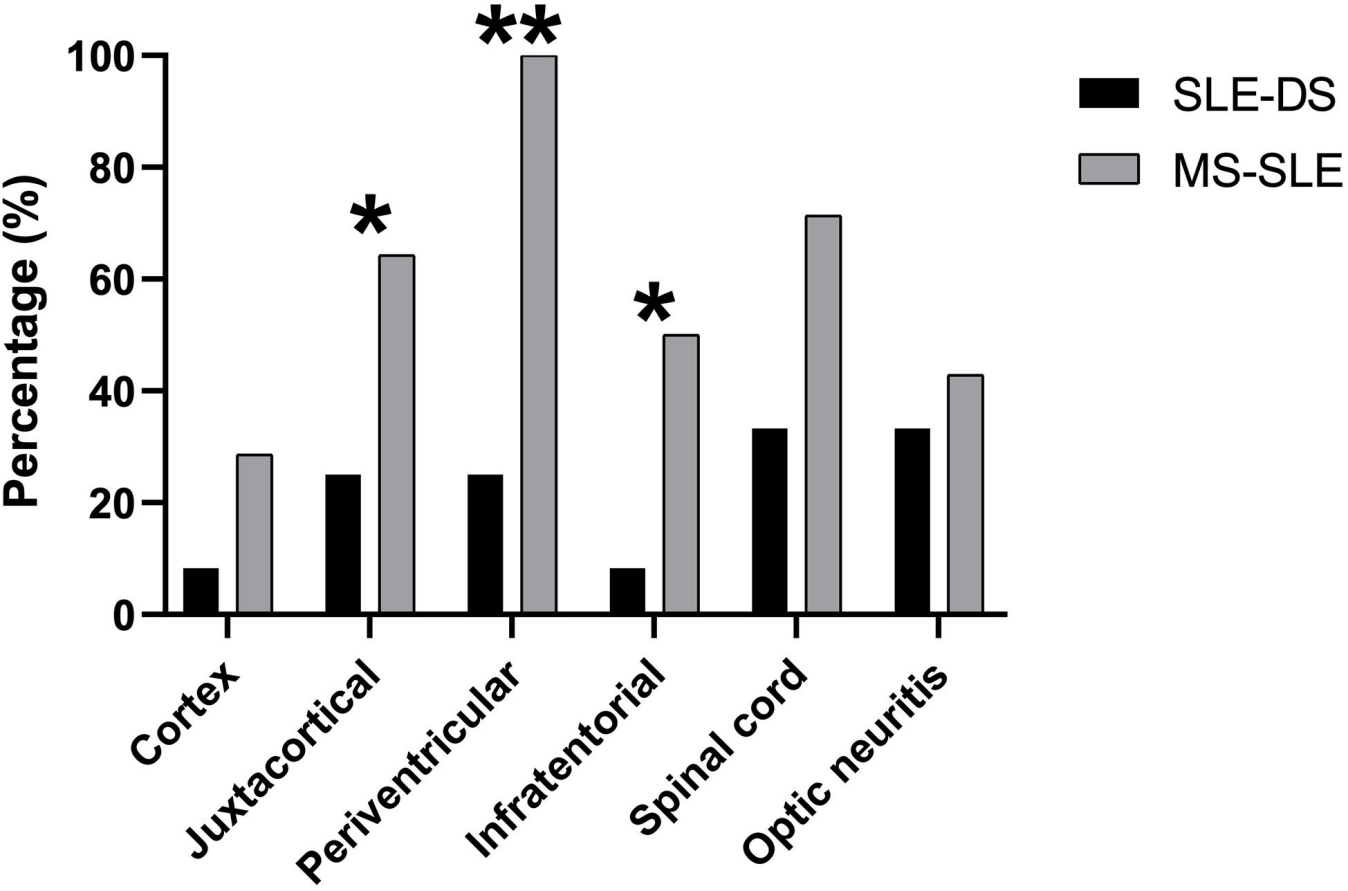
Anatomical distribution of CNS lesions on brain, spinal and orbital MRI, in patients with SLE-demyelinating syndrome and patients with overlap SLE-MS. *p < 0.05, **p < 0.01

Regarding MRI findings, both the brain and spinal cord were more likely to be affected in overlap SLE-MS patients, while optic nerve involvement was similarly affected in the two groups (Supplementary Table 4). A detailed anatomical distribution of CNS lesions is shown separately for patients with SLE-DS and SLE-MS in Figure 1. As expected, the former were less likely to exhibit brain lesions in the spinal cord, infratentorial, periventricular, and juxtacortical regions. More importantly, only a single brain region was affected in 9/12 patients with SLE-DS (75%), contrary to all SLE-MS patients who had multiple affected brain regions.

### Overlap SLE-MS Is Associated With More Relapses and Worse Outcome

At the end of our observational period, overlap SLE-MS was associated with an increased likelihood of relapses (OR 18.2, 95% CI: 1.76–188). Specifically, only 5/12 patients with SLE-DS exhibited a relapse, while 92.9% of SLE-MS patients experienced at least one relapse (*p*-value = 0.004, Supplementary Table 5).

**Figure 2:**
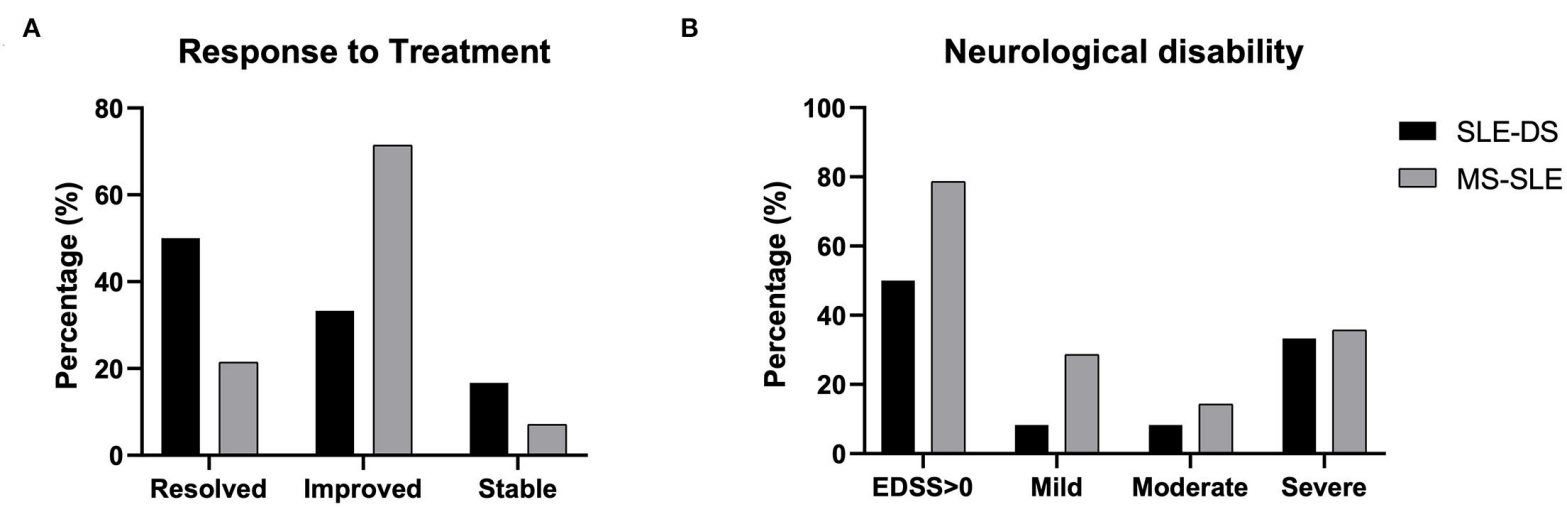
Disease- and treatment-related outcomes at the most recent-follow up visit in patients with SLE-demyelinating syndrome and patients with overlap SLE-MS. **(A)** Response to treatment categorized as i) complete resolution of neurologic symptoms, ii) partial improvement of neurologic symptoms, or iii) no improvement (stable). **(B)** Neurological disability, as measured by the expanded disability status scale. Mild disability: EDSS ≤ 2; moderate disability: EDSS 2–4; severe disability: EDSS >4. No significant differences were captured between the two groups.

Disease-related outcomes, including response to treatment and neurological disability at the most recent visit, are shown separately for patients with SLE-DS and SLE-MS overlap in Figure 2. Importantly, only 3/26 patients (11.5%, 2 with SLE-DS and one with SLE-MS) did not respond to treatment and their neurological symptoms remained unaltered. The majority patients with of SLE-MS (71.4%) showed a partial response of their neurologic symptoms, while complete response was achieved in 3 patients (21.4%). On the contrary, demyelinating episodes in SLE-DS patients resolved completely in 50% (6/12), while 4/12 (33.3%) showed only partial improvement (Figure 2A).

Finally, neurological disability, as measured by EDSS, is shown in Figure 2B. Patients with SLE-DS were less likely to exhibit neurological deficits (EDSS >0) at the end of follow-up, as compared to patients with SLE-MS (50 vs. 78.6%, respectively, *p* = ns). Importantly, approximately half of the patients in each group had moderate to severe neurological deficits at the last evaluation.

## Discussion

The occurrence of DS in a patient with SLE represents a diagnostic and therapeutic challenge. Whether such a patient has a neurologic manifestation of their systemic autoimmune disease or two different diseases, is not only a question of theoretical value; on the contrary, drugs used to treat SLE and MS differ significantly. Furthermore, some therapies, like interferon-based regimens used in MS, may trigger disease flares in patients with lupus, of whom ~75% exhibit a strong interferon signature (10, 11). To this end, in this study, we aimed to provide a detailed longitudinal evaluation of demyelination presenting in patients with SLE to assess its natural course and identify potential factors that can predict which patients will eventually evolve to frank MS.

Very few studies to date have attempted to describe DS in the context of SLE in detail. Piga et al. (12) performed a systematic literature review, including patients from their own cohort, to identify a total of 104 SLE patients with DS and calculated an estimated prevalence of 1.3%. In this very comprehensive review, the authors opted to include NMO and NMOSD, which comprised more than 60% of patients, as SLE-related demyelination. Nevertheless, due to the high specificity of anti-aquaporin-4 antibodies for NMOSD, (13, 14), the current consensus argues that when the latter occur in patients with SLE, this most probably represents the coexistence of two autoimmune conditions (15). For this reason, in our study, we excluded patients with NMOSD. We also chose to exclude patients with longitudinal myelopathy attributed to SLE (i.e., anti-aquaporin-4 negative). Although longitudinal myelopathy can be considered a demyelinating condition, it also stands alone as a distinct neuropsychiatric manifestation of SLE. Thus, as the purpose of our study was the differentiation between SLE and MS, we felt that lupus myelopathy does not pose the same diagnostic challenges in patients with a demyelinating syndrome compatible with MS. Finally, in the study by Piga et al. another 27.9% of patients had a CIS. Information on the duration of follow-up was not available, but it would be interesting to know whether at least a proportion of patients with CIS fulfilled criteria for MS in the course of time.

Fourteen patients in our cohort fulfilled the criteria for MS at the most recent-follow up, thus labeled as SLE-MS overlap. Although the segregation of more than one autoimmune disease may occur in the same individual, the coexistence of MS and SLE has only rarely been reported, mainly in case reports. In a previous work from a different SLE cohort (the “Leto” cohort in Crete) (16), we have described another case series of nine patients who fulfilled the criteria for both the diseases (3) We observed similar patient characteristics in both case series. Specifically, overlap patients tended to have a relatively mild SLE phenotype, with no major extra-CNS organ involvement, which did not necessitate intensive immunosuppressive treatment. Contrary, MS tends to follow a relapsing–remitting course, with a variable accumulation of disability, and its severity usually dictates the choice of immunomodulating agents.

Identification of clinical or laboratory features early in the course of a DS that would help predict which patients will eventually evolve to MS would be very helpful. In terms of clinical presentation, no rheumatic or neurologic manifestation was significantly different between SLE-DS and overlap SLE-MS patients. Contrary, we confirmed the diagnostic value of lumbar puncture and CSF analysis in the work-up of patients with demyelination. Both an elevated IgG index and, especially, the presence of type 2 oligoclonal bands was strongly predictive of a final MS diagnosis since both were significantly more common in these patients compared to SLE-DS. This observation corroborates the most recent update of the diagnostic criteria for MS, wherein the presence of unmatched CSF oligoclonal bands permits the diagnosis of MS, even without proven dissemination in time clinically or on MRI (17). Although the 2017 criteria have been criticized by some for lower specificity, our findings support a low threshold for CSF analysis in patients presenting with DS.

The burden of MRI lesions in the CNS was also significantly different between SLE-DS and overlap SLE-MS patients, both in terms of number and location of lesions. Overlap patients tended to have lesions in locations typical for MS, including the infratentorial region and the spinal cord. By contrast, patients with SLE often had lesions only in a single brain territory. Dissemination of CNS lesions in space is a hallmark of MS, which tends to accrue over time and be associated with progressive neurologic disability (18). Accordingly, overlap SLE-MS patients in our cohort accumulated significantly more neurologic damage until the end of follow-up, as measured by the EDSS.

Our study has several limitations. Firstly, similarly to the criticism of the aforementioned systematic review, the relatively short follow-up (little over 3 years) of our study cannot exclude that lupus patients with a CIS in our cohort will not evolve into definite MS in the future. Also, our study did not aim to address the issue of therapy of demyelination in the context of SLE, either SLE-DS or SLE-MS. In this regard, one cannot exclude that the natural history of the demyelinating syndrome could have been influenced by the administration of immunosuppressive or disease-modifying therapies. Along the same lines, in the era of current biologic therapies, demyelination may occasionally occur as a side-effect of medications (19, 20). Nevertheless, in our series, only seven patients with SLE had received immunosuppressive treatment prior to the first occurrence of a demyelinating event (glucocorticoids, hydroxychloroquine, methotrexate, azathioprine, and belimumab) None of these drugs has been linked to demyelinating episodes as a side- effect.

In conclusion, we present one of the few studies with a detailed description of DS in the context of SLE and, for the first time, a longitudinal assessment of DS occurring in patients with SLE, either prior to or following the diagnosis of lupus. We found that more than 50% of these patients are finally diagnosed with MS, while demyelination in the context of SLE follows a more benign course compared to a frank SLE-MS overlap. More importantly, further extension of follow-up in these patients will ascertain whether the remaining SLE-DS patients evolve to MS, or whether SLE-DS is indeed a *bona fide* syndrome of NPSLE.

## Data Availability Statement

The raw data supporting the conclusions of this article will be made available by the authors, without undue reservation.

## Ethics Statement

The studies involving human participants were reviewed and approved by Attikon University Hospital Ethics Committe. The patients/participants provided their written informed consent to participate in this study.

## Author Contributions

DN and DK examined patient files and identified candidate patients for the study and DN drafted the manuscript. MP reviewed all brain imaging data. NK, AP, PG, and AG contributed to patient recruitment and data entry. KV, DB, and AF conceived the study, confirmed final patients‘diagnoses, and critically revised and oversaw the study and the writing. All authors contributed to the article and approved the submitted version.

## Funding

This work was funded in parts by the Hellenic Society of Rheumatology, the Foundation for Research in Rheumatology (FOREUM), the Greek General Secretariat of Research and Technology ‘Aristeia' action of the Operational Program ‘Education and Lifelong Learning' (cofounded by the European Social Fund and National Resources, Aristeia I 2344 to D.T.B), the European Research Council (ERC) under the European Union's Horizon 2020 Research and Innovation Programme (grant agreement no. 742390), and the SYSCID (A Systems Medicine Approach to Chronic Inflammatory Diseases) under the European Union's Horizon 2020 Research and Innovation Programme (grant agreement no. 733100).

## Conflict of Interest

The authors declare that the research was conducted in the absence of any commercial or financial relationships that could be construed as a potential conflict of interest.

## Publisher's Note

All claims expressed in this article are solely those of the authors and do not necessarily represent those of their affiliated organizations, or those of the publisher, the editors and the reviewers. Any product that may be evaluated in this article, or claim that may be made by its manufacturer, is not guaranteed or endorsed by the publisher.
