# Supplementary material for: Demyelinating Syndromes in Systemic Lupus Erythematosus: Data From the “Attikon” Lupus Cohort
Source: Front Neurol. 2022 May 11;13:889613. doi: 10.3389/fneur.2022.889613 (PMC9131105; doi:10.3389/fneur.2022.889613)
Supplement: Supplementary file 1 [file Data_Sheet_1.docx]

Supplementary Material

**Supplementary Table 1. Demographic characteristics of SLE patients with demyelinating syndromes compared to the whole “Attikon” lupus cohort**

|  | **Total (n=26)** | **SLE-DS (n=12)** | **MS-SLE (n=14)** |
| --- | --- | --- | --- |
| Female sex, n | 25/26 | 11/12 | 14/14 |
| Age at SLE diagnosis, mean (SD), years | 46.92 (12.3) | 47.41 (12.9) | 46.5 (12.2) |
| SLE disease duration, median, IQR (months) | 60 (52) | 47.5 (85) | 60 (39.5) |
| Delay in MS diagnosis >2 months, n | NA | NA | 6/14 |
| Early MS diagnosis | NA | NA | 8/14 |
| Relapsing-remitting MS | NA | NA | 12/14 |
| Secondary progressive MS | NA | NA | 2/14 |
| Demyelination before SLE Diagnosis | 13/26 | 4/12 | 9/14 |
| Demyelination after SLE diagnosis | 7/26 | 2/12 | 5/14 |
| Demyelination at SLE diagnosis | 6/26 | 6/12 | 0/14 |

MS: multiple sclerosis; SLE: Systemic lupus erythematosus; DS: demyelinating syndrome NA: Not applicable

**Supplementary Table 2. Clinical features and autoantibodies in SLE patients with demyelinating syndromes**

| **Clinical manifestations** | **SLE-DS**  **(n=12)** | **MS-SLE (n=14)** | **P-value** |
| --- | --- | --- | --- |
| Acute cutaneous lupus, n(%) | 11(91.7) | 13(92.9) | ns |
| Malar rash, n(%) | 7(58.3) | 10(71.4) | ns |
| Photosensitivity, n(%) | 4(33.3) | 4(28.6) | ns |
| Chronic cutaneous lupus n(%) | 0(0) | 2(14.2) | ns |
| Oral ulcers, n(%) | 1(8.3) | 5(35.7) | ns |
| Non-scarring alopecia, n(%) | 4(33.3) | 2(14.3) | ns |
| Inflammatory arthritis, n(%) | 11(91.7) | 13(92.9) | ns |
| Serositis, n(%) | 2(16.7) | 0(0) | ns |
| Lupus nephritis, n(%) | 0(0) | 1(7.1) | ns |
| Neuropsychiatric events^&^, n(%) | 2(16.7) | 4(28.6) | ns |
| Leukopenia, n(%) | 0(0) | 4(28.6) | ns |
| Thrombocytopenia, n(%) | 0(0) | 0(0) | ns |
| Hemolytic anemia, n(%) | 0(0) | 0(0) | ns |
| Fever, n(%) | 1(8.3) | 1(7.1) | ns |
| Raynaud’s, n(%) | 4(33.3) | 4(28.6) | ns |
| **Autoantibodies** | | | |
| ANA, n(%) | 12(100) | 12(85.7) | ns |
| Anti-dsDNA, n(%) | 4(33.3) | 3(21.4) | ns |
| Anti-Smith, n(%) | 1(8.3) | 1(7.1) | ns |
| Low C3 and/or C4, n(%) | 6(50) | 6(42.9) | ns |
| Anti-SSA, n(%) | 4(33.3) | 3(21.4) | ns |
| Anti-SSB, n(%) | 1(8.3) | 2(14.3) | ns |
| Anti-phospholipids, n(%) | 1(8.3) | 2(14.3) | ns |
| Anti-RNP, n(%) | 2(16.7) | 1(7.1) | ns |

**Supplementary Table 3. Neurologic clinical features of patients with SLE-demyelination and MS-SLE**

|  | **Total (n=26)** | **SLE-DS**  **(n=12)** | **MS-SLE (n=14)** | **P-value** |
| --- | --- | --- | --- | --- |
| Sensory, n (%) | 12(46.2) | 5(41.7) | 7(50) | Ns |
| Motor, n (%) | 7(26.9) | 4(33.3) | 3(21.4) | Ns |
| Optic neuritis, n(%) | 9(34.6) | 4(33.3) | 5(35.7) | Ns |
| Diplopia, n(%) | 1(3.8) | 0(0) | 1(7.1) | Ns |

**Supplementary Table 4. Location of CNS lesions in patients with SLE-demyelination and MS-SLE**

|  | **Total (n=26)** | **SLE-DS**  **(n=12)** | **MS-SLE (n=14)** | **P-value** |
| --- | --- | --- | --- | --- |
| Brain, n(%) | 20 (76.9) | 6 (50) | 14 (100) | NA |
| Optic nerve, n(%) | 10 (38.5) | 4 (33.3) | 6 (42.9) | Ns |
| Spinal cord, n(%) | 14 (53.8) | 4 (33.3) | 10 (71.4) | 0.052 |

NA: not applicable

**Supplementary Table 5. Relapses in patients with SLE-demyelination and MS-SLE**

|  | **Total (n=26)** | **SLE-DS**  **(n=12)** | **MS-SLE**  **(n=14)** | **P-value** |
| --- | --- | --- | --- | --- |
| Relapses > 0, n(%) | 18 (69.2) | 5 (41.7) | 13 (92.9) | **0.004** |
| Mean (SD) | 2.1 (5.2) | 1.0 (2.9) | 3.1 (5.2) | **0.006** |
| Median (IQR) | 1 (4) | 0 (1) | 3.5 (4) |  |
